# Low temperature preparation of diopside nanoparticles: in-vitro bioactivity and drug loading evaluation

**DOI:** 10.1038/s41598-023-43671-0

**Published:** 2023-09-28

**Authors:** Ava sobhani, Esmaeil Salimi

**Affiliations:** Faculty of Chemical and Materials Engineering, Shahrood University of Technology, P. O. Box: 3619995161, Shahrood, Iran

**Keywords:** Biomaterials, Nanoscale materials

## Abstract

Bioactive diopside (CaMgSi_2_O_6_) nanoparticles have recently gained potential usefulness as bone replacement materials and nano vehicles for delivering therapeutics. The structural characteristics of this ceramic have found to be a key factor in bone bonding ability. To attain the desired product for 100% clinical success, it is important to realize the relationship between structure and biological activity. Synthesis of these nanoparticles via the solid-state method has been regarded as a low-cost and easy process in large-scale, but time consuming reactions and high temperature (≈ 1400 °C) are required. On the other side, the wet chemistry can overcome these drawbacks, whereas the presence of byproducts in the final powder has limited this method in large-scale production. The present document has represented a simple, fast and one-pot sol–gel approach for the synthesis of highly pure diopside nano-powders (< 20 nm) by using not-expensive precursors. Calcination of the obtained powder has been conducted at various temperatures (700, 1000 and 1200 °C). The physicochemical and microstructural properties of the products have been characterized by XRD, FTIR, FESEM and TEM. Moreover, the impact of the crystallinity on the bioactivity, drug loading capacity and drug release behavior of the synthesized nanoparticles have been investigated here for the first time. The in-vitro bioactivity results of the prepared diopside samples in a simulated body fluid (SBF) at 37 °C revealed the higher capability of the sintered sample to deposit calcium phosphate, compared with the amorphous one. High quantity of gentamicin (around 10 µg) could attach to the surface of 1 miligram of the sintered diopside during the early stages of contact (3 h), suggesting the potential use of diopside as a new class of nano-vehicles for antibiotics. The release behavior indicated a sustained release of gentamicin (80%) after 24 h. In conclusion, diopside nanoparticles can be a promising candidate as a drug-vehicle for bone filling, implant coating or bone cement applications.

## Introduction

Finding a synthetic bone substitute to act similar to autologous bone grafts remained a challenge in bone tissue engineering. Osteogenic stimulation^1^ and bone bonding abilities^2^ are among the attractive biological properties of bioglasses that made them in the center of attention. During the last years, a number of glasses, ceramics, glass–ceramics and composites^3–5^ have been employed in biomedical fields. These compounds were famous for their bioactivity and formation of chemical interactions with the surrounding bone via the generated mediate biological apatite layer. Previous studies have indicated the superior bioactivity of the calcium-silicate glass–ceramics such as wollastonite (CaSiO_3_)^6^ and diopside (CaMgSi_2_O_6_)^7^. Moreover, Kokubo et al.^8^ have specified the rapid precipitation of an hydroxyapatite (HA) layer on the surface of the glasses placed in the simulated body fluid (SBF). Several reports have evaluated the biological features of diopside in powder or bulk form. According to these reports, calcium silicate-based materials have exhibited brilliant bioactivity and biocompatibility providing high capacity for hard tissue engineering such as biocement, bone graft and metal implant coating^9^. Sadeghzade et al.^10^ have applied a thin layer of polycaprolactone polymer on the surface of diopside/baghdadite composite scaffolds to decrease the degradation rate and control the apatite formation ability. By using this strategy, they could also improve the attachment and proliferation of the bone marrow stem cells.

The calcium-magnesium silicate ceramic diopside (CaMgSi_2_O_6_), which is characterized by high bioactivity, biocompatibility, and better mechanical properties than hydroxyapatite, has recently been studied for use in regenerative medicine^11^. The high biocompatibility of diopside is attributed, in particular, to its ability of bio-mineralization^12^.

Solid-state reactions have been traditionally utilized for the synthesis of diopside powder^13^. Although preparation of nano-scale diopside via this process is difficult, because of the high temperatures and long reaction time that results in the growth of grains. But, the value of this method in large-scale production is still evident. Recently, nano-powders have attracted much attention due to their broad applications in medicine, electronic and etc. Chemical approaches such as hydrothermal^14^, sol–gel^15^, precipitation^16^ and also natural wastes such as rice husk ash and eggshells^17^ have been employed for the synthesis of diopside at low temperatures. Venkatraman et al.^18^ have prepared the pure phase of monticellite and diopside from bio-waste by using a self-propagating auto combustion route. They have reported that the phase formation temperature can influence the grain size. In another study^19^, different content of diopside have been added to forsterite to obtain porous scaffolds via space holder method. Diopside can reduce the sintering temperature and therefore hinder grain growth. Addition of 10 wt% of diopside could dramatically enhance the bioactivity and biodegradability. Moreover, the chemical composition of this ternary oxide (CaO–MgO–SiO_2_) system had a remarkable effect on bioactivity, resorbability and dissolution properties. Fine powders as well as porous or dense films, bulks and fibers products with good sinter-ability can be obtained by sol–gel method^19–23^. Moreover, complex products with high homogeneity have been prepared at low temperatures. In this method, metal alkoxides have been usually used as precursors. Metal alkoxides have different hydrolysis rate and therefore, suggesting a synthesis method with repeatability is not easy^24^. Instability of most of the metal alkoxides in the air has limited their handling in the preparation procedure. So, it is expected that utilizing metal salts along with metal alkoxides as precursors for the synthesis of diopside via the sol–gel method can overcome the mentioned weakness.

On the other hand, combination of drug delivery technology with bone fillers is an interdisciplinary area that can shorten the treatment period. Antibiotics such as gentamicin (GEN) are generally used to prevent the bone infections after surgery^20^. However, the cells viability may be endangered when subjected to high dosage of GEN. So, in order to overcome this problem and reduce the therapeutic dosage, designing a drug delivery system has been suggested. Development of a local delivery system can control the drug release profile and inhibit the burst release. A variety of bioceramics and composites have been used as targeted drug delivery systems^21–23^. Kudinova et al.^24^ have loaded the antibacterial enzyme lysostaphin on diopside powder and studied the antibacterial and antibiofilm properties of such material to probe the utility of this approach for diopside-based prosthetic materials. Porous diopside microspheres were employed as a carrier for dexamethasone. It has been found that the porosity of spheres may affect the drug delivery performance^25^. Since the crystallinity of a material can influence the porosity and surface properties, the present study has investigated the synthesis of diopside nanoparticles via a simple procedure. The effect of bioglass crystallinity on adsorption and desorption of gentamicin has been also studied by using the amorphous and crystalline (sintered at 1000 °C) diopside powders.

## Materials and methods

### Materials

Magnesium chloride (MgCl_2_) and calcium nitrate Ca(NO_3_)_2_·4H_2_O, tetraethoxysilane (TEOS) and ethanol were supplied by Merck. Gentamicin sulfate (GEN) was purchased from an Iranian pharmaceutical manufacturing. All chemicals were of the analytical grade and used without purification.

### Preparation of diopside

First of all, 0.125 mole of MgCl_2_·6H_2_O and 0.125 mole of Ca (NO_3_)_2_·4H_2_O have been dissolved in ethanol, stirred for 1 h. Then, 0.250 mole of TEOS has been added to the above medium and stirred for some hours. The resultant gel has been dried in an oven at 110 °C for 1 day. Followed by calcination of the dried powder at 700, 1000 and 1200 °C for 2 h in a furnace with 10 °C min^−1^ heating rate.

### Characterization

The prepared powders were grinded by a mortar, mixed with KBr and analyzed by Fourier transform infrared spectra (FTIR) spectroscopy to identify the functional groups. The X-ray diffractometer (PW1730, PHILIPS X′Pert Pro, Netherland) was used to identify the crystallinity of the prepared samples. The spectra were recorded in the 2θ range from 10° to 80° with CuKα (λ = 1.5406 Å) as the radiation source at a current of 30 mA and with an accelerating voltage of 40 kV. The morphology and microstructure of the prepared powders were observed by field emission scanning electron microscopy (FESEM, Zeiss HV-300-Germany) associated with energy dispersive X-ray analyzer (EDX, Oxford AZtec1-England) and transmission electron microscopy (TEM, CM120, Netherland).

### In-vitro bioactivity study

The bioactivity of the prepared samples was evaluated upon immersion in simulated body fluid (SBF) for 21 days. The SBF was prepared based on the usual kokubo method^26^. Formation of an apatite layer on the samples surface after 21 days indicated the level of bioactivity, which was analyzed by SEM and EDX.

### Gentamicin loading and release study

Gentamicin sulfate (BioBasic Co. Canada), a low cost and effective antimicrobial drug was selected as a model antibiotic. 10 mg of drug was dissolved in 10 mL of PBS and used as the stock solution for the loading test. The stock solution was diluted to reach the drug concentration of 100 µg mL^−1^. Different quantity (10 and 20 mg) of diopside powder were suspended in the drug solution into a 10 mL tube and agitated for 2 days. 1 mL of supernatant was collected at different time intervals to analyze quantitatively. The amount of the adsorbed gentamicin on the surface of the diopside was quantified indirectly by measuring the concentration of the drug in the solution before and after the loading test.

The in-vitro drug release investigation was performed by placing a predetermined amount of the drug loaded diopside particles in different tubes containing the PBS solution at 37 °C and pH = 7.4, and shaken gently. In order to quantify the released gentamicin, different samples were prepared for different time points, which analyzed by UV–Vis spectrophotometry at λ = 201 nm. The drug release experiment was performed in triplicate and the percentage of the released gentamicin was plotted as a function of time.

## Results and discussions

**Figure 1:**
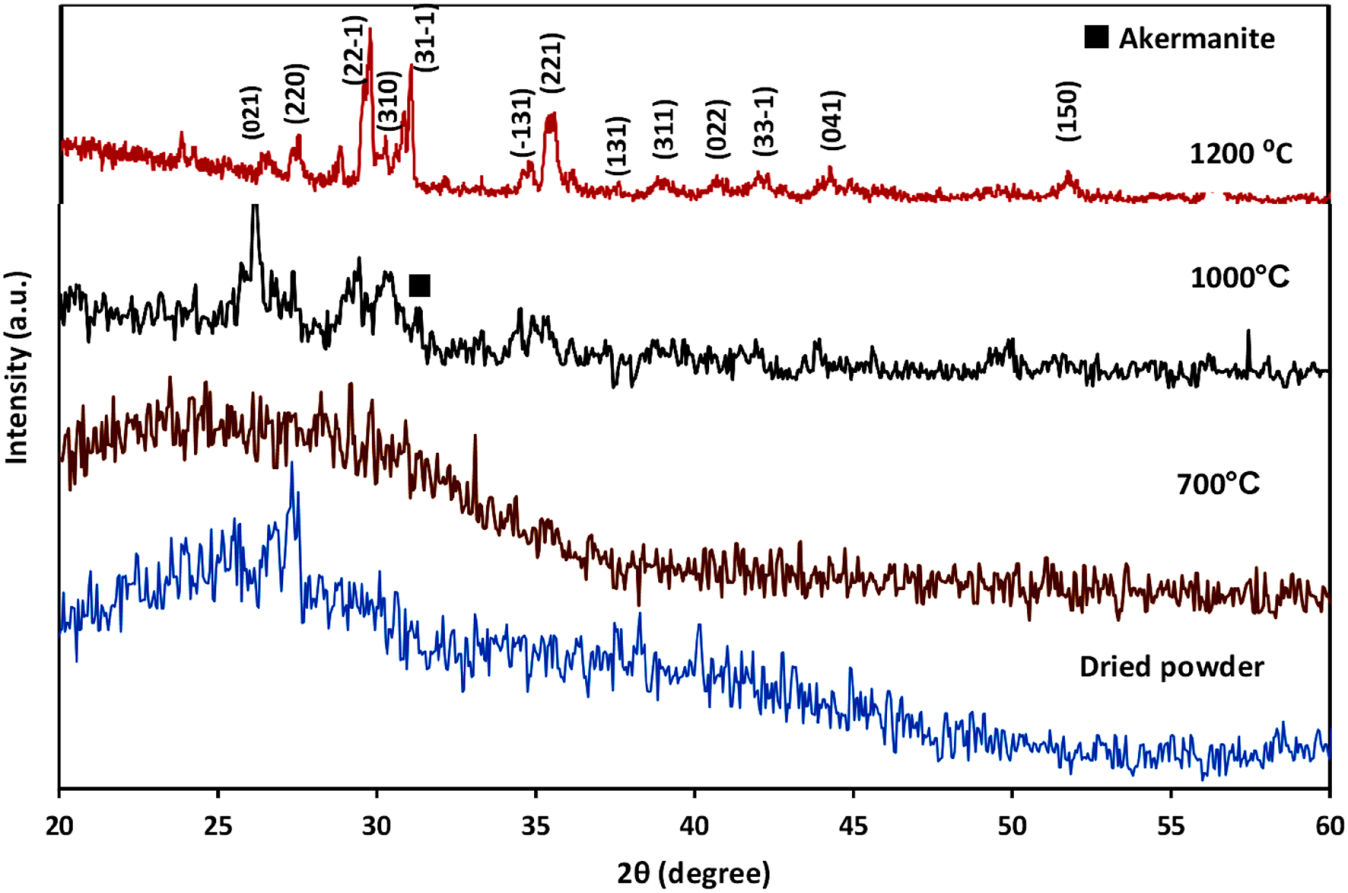
X-ray diffraction patterns of the synthetic diopside after heating at different temperatures.

The X-ray diffraction was used to identify the crystalline phases. Figure 1 represented the XRD patterns of the prepared powders heated at various temperatures (700–1200 °C). The XRD pattern of the sample sintered at 700 °C did not present the characteristic peaks of diopside. It has been reported that agglomerated structure of the bioglass particles could limit the complete transformation of C–S–H into wollastonite bioglass, resulted in the presence of amorphous structure along with the crystalline phase at this temperature^27^. Whereas, the peaks intensity of the gel powder sintered at higher temperatures was increased, indicated the crystallized diopside phase. Similar observations were also reported by Iwata et al.^28^ and Yamamoto et al.^15^, where the single phase of diopside was crystallized after heat treatment above 800 °C. Interestingly, the type of precursor can affect the calcination temperature. Choudhary et al.^29^ reported that the calcination temperature for the crystalline diopside prepared by eggshell by using urea as a fuel was optimized at 800 °C which was found to be very low when compared with our observation and also earlier reports^30^. It has been found that pure phase of diopside could be achieved by calcining the powders around 1100–1300 °C^31, 32^. Akermanite and monticellite were usually observed as the secondary phases in the time of diopside synthesis. The present results disclosed that calcination of the sample at 1000 °C resulted in the formation of diopside as the major phase and akermanite as the secondary phase. Upon increasing the calcination temperature up to 1200 °C, akermanite peak was completely removed and single phase diopside was formed. Table 1 shows the interplanar d-spacings and positions (2θ) of the diffraction peaks related to the X-ray patterns of the calcined samples. The computed d-spacings were in close agreement with the data of ICDD card of 01-075-1092, with no extra impurity peaks. The Debye-Scherer equation was used to calculate the crystallite size from full width at half maximum value in the XRD pattern^33^. The crystallite size value was found around 5.3–13.8 nm, by selecting the intense diffraction peak at 2θ = 26.

**Table 1:** The position and characteristics of the main XRD peaks of diopside.

Pos. [°2Th.]	Height [cts]	FWHM Left [°2Th.]	d-spacing [Å]	Rel. Int. [%]	Tip width	Matched by
26.0219	86.45	0.5904	3.42429	100.00	0.7085	01-075-1092
29.8314	63.61	1.5744	2.99512	73.57	1.8893	01-075-1092
34.9491	55.19	1.1808	2.56738	63.84	1.4170	01-075-1092
49.6807	29.33	0.7872	1.83516	33.92	0.9446	01-075-1092

**Figure 2:**
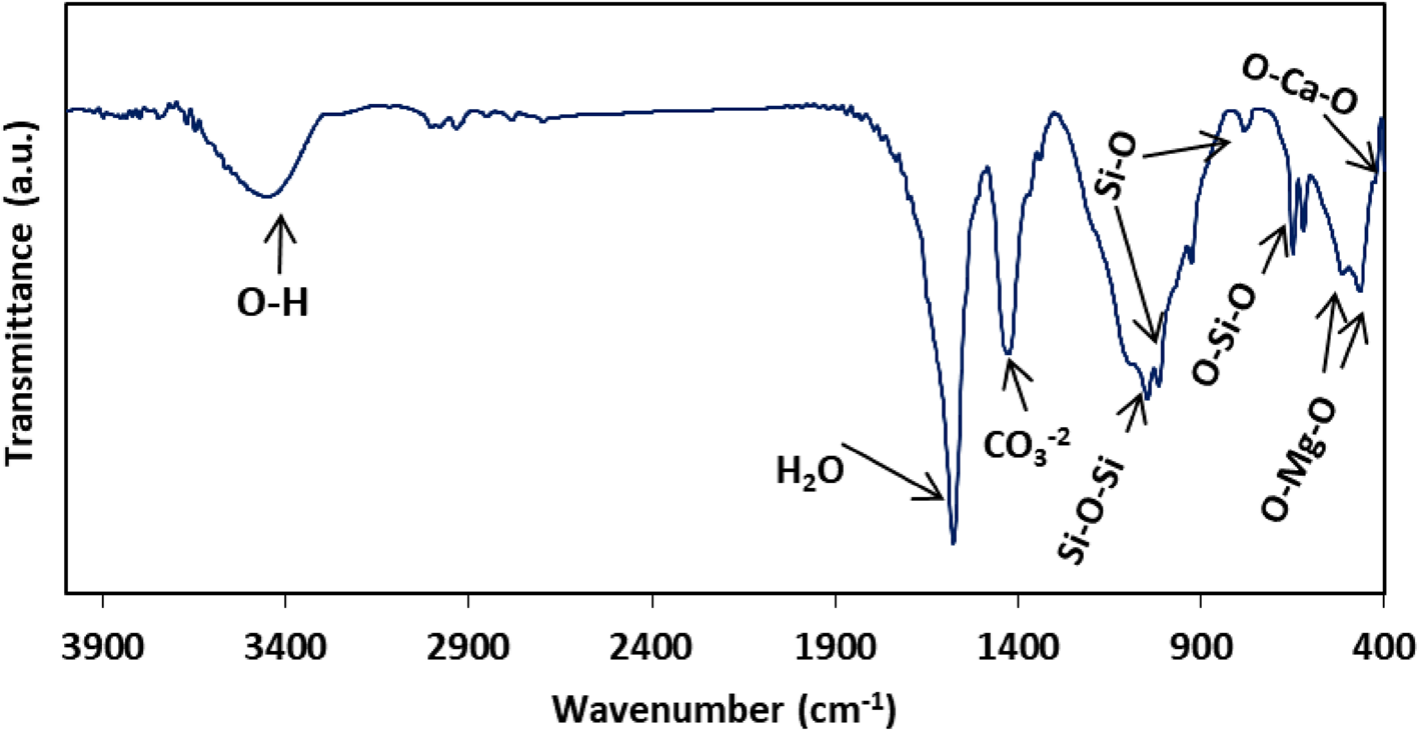
FTIR spectrum of the prepared diopside.

The Fourier transformed infrared spectroscopy was used to analyze the functional groups in the final products structure, as recorded in Fig. 2. Based on the reports, the absorption bands at around 619, 648 and 1046 cm^−1^ could be related to the Si–O–Si and O–Si–O bonds^34–36^. Decomposition of silica occurs during heat treatment, followed by incorporation of Ca^+2^ inside the silica framework; Resulted in the emergence of the Si–O–Ca non-bridging oxygen bonds, which could be attributed to the appeared vibration around 1015 cm^−1^. Usually, the non-bridging bending vibrational modes of O–Ca–O could be found around 420 cm^−1^, which unfortunately was not clear in our data. The O–Mg–O non-bridging bending modes was appeared in the range of 455 to 503 cm^−1^^37^. Within the gel, the sharp intense peak at 1576 cm^−1^ and the broad band at 3445 cm^−1^ symbolized to the bending and stretching vibrations of absorbed water molecules and OH groups. The observed pattern of the functional groups was in agreement with the earlier findings^28, 37^.

The scientific literature stated that vibrations of C–O bonds in CO_3_^2−^ groups can be detected at approximately 1450, 1050, 820, and 740 cm^−1^, including asymmetric and symmetric stretching, as well as out-of-plane and in-plane bending vibrations. These vibrations were produced due to contamination with atmospheric CO_2_ during the synthesis process^38^. Additionally, previous literatures have reported a distinct peak at around 1300–1400 cm^−1^ associated with the symmetric stretching vibrations of NO^-^_3_ groups, which originate from nitrate-type precursors used in the precursor solution, since the complete decomposition of nitrate occurs at higher than 600 °C^29, 39^. So, it is also possible that the observed peak around 1460 cm^−1^ was actually the overlap of C–O and N–O bands.

**Figure 3:**
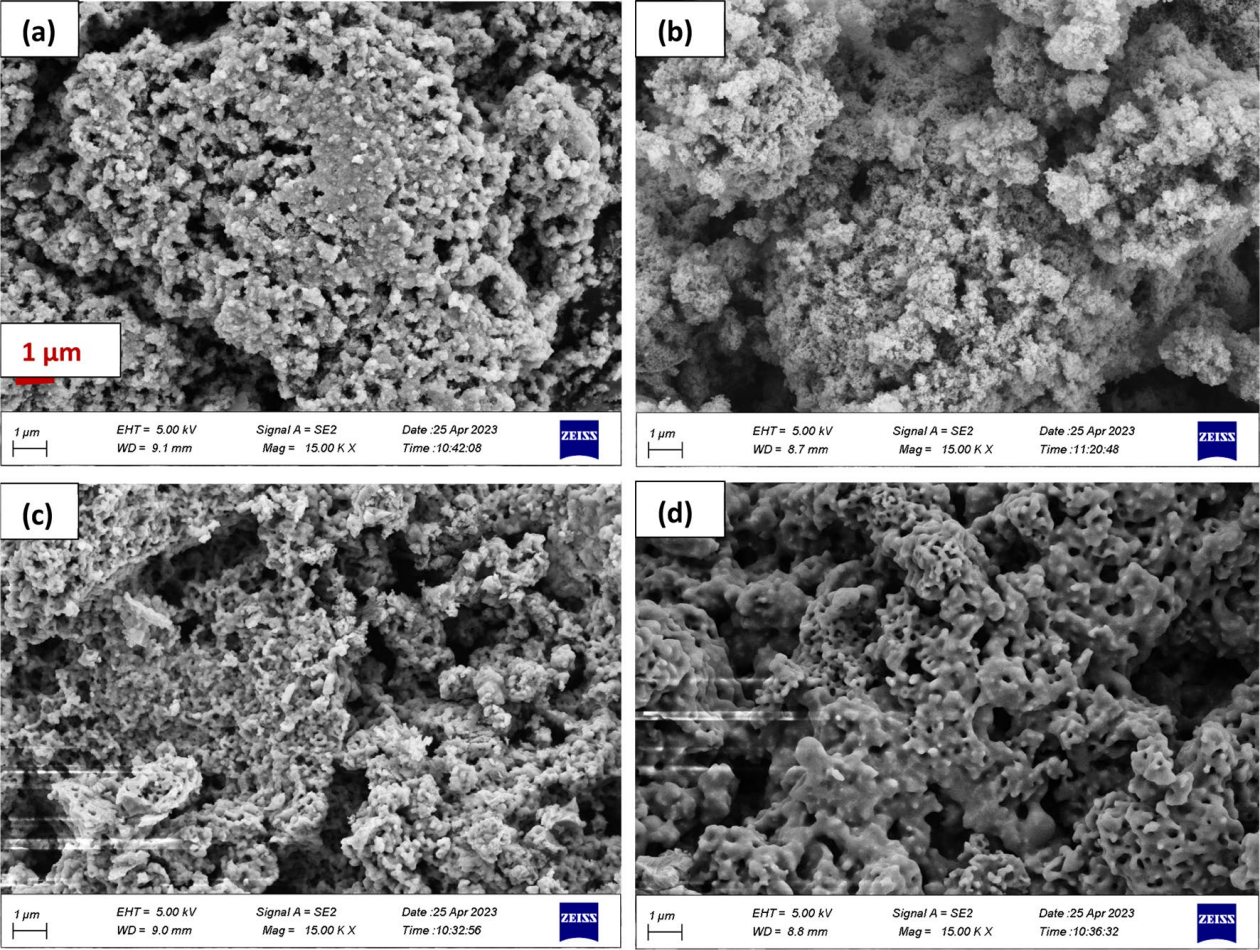
FESEM images of the (**a**) prepared diopside nanoparticles, and sintered powder at (**b**) 700, (**c**) 1000 and (**d**) 1200 °C.

**Figure 4:**
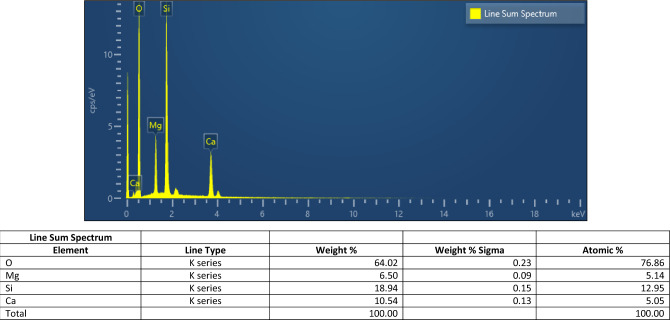
The EDX data of the prepared diopside.

According to the literatures, the texture of bioglass can affect its bioactivity and also crystallization and porosity of the specimens can enhance the rate of apatite materialization^40, 41^. So, the morphology of the obtained materials were carefully studied by the FESEM and represented in Fig. 3. The micrographs of the calcined diopside at 700 °C (Fig. 3b) exhibited the agglomeration of small particles consist of sphere-like dioside crystals. The powder sintered at higher temperature 1200 °C had been almost melted and separate particles were not discernible. The accompanied EDX data (Fig. 4) revealed the elemental analysis of the synthesized diopside nanoparticles. The atomic percent of Ca was similar to Mg, where the Si quantity was almost twice of Ca, which was in accordance with the diopside chemical formula.

**Figure 5:**
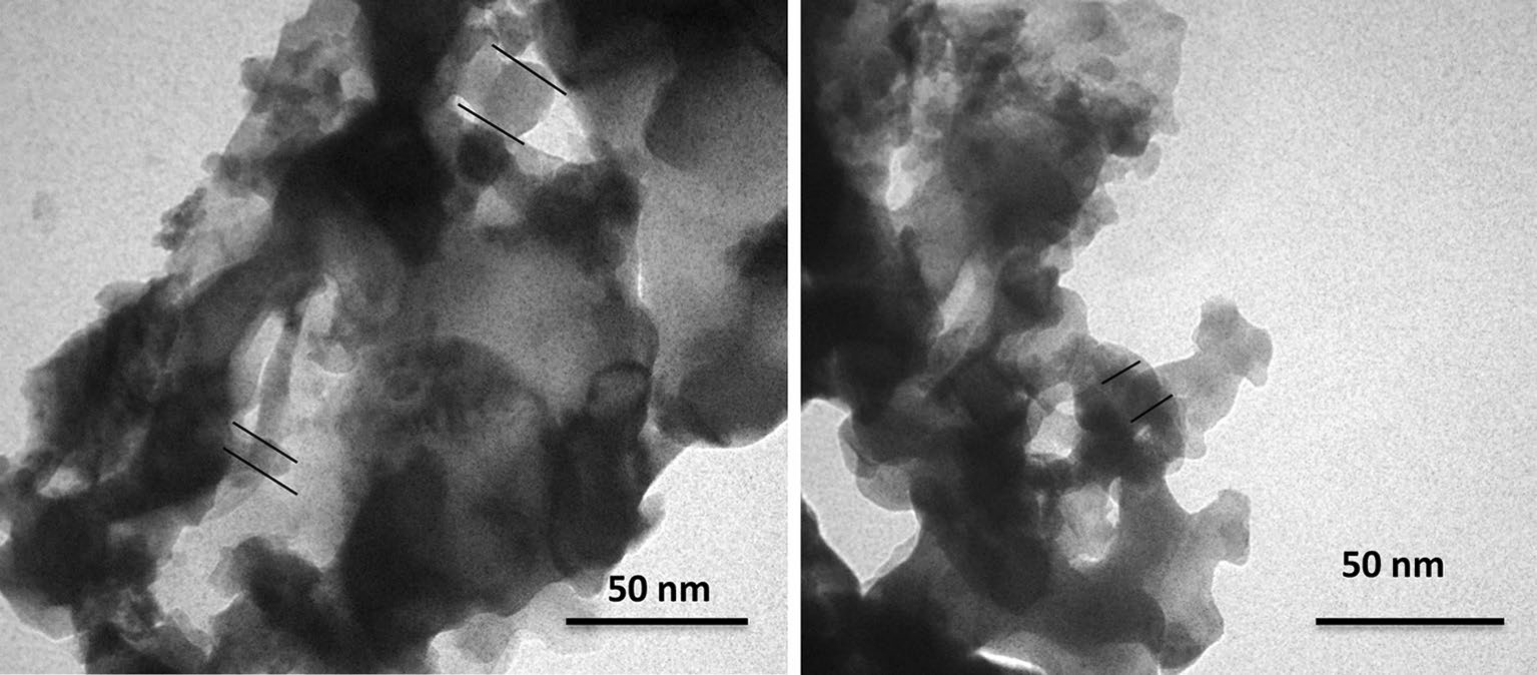
TEM images of the synthesized diopside nanoparticles.

The TEM micrographs in Fig. 5 showed particles of < 20 nm agglomerated and stacked to create even larger objects. Moreover, some organic residue could be seen on the surface of particles, which affected the clarity of the pictures. The particles had elongated shapes and in some cases, they showed sharp edges. The flaky shape of aggregates has hindered the clear observation of the particles, separately. The size discrepancies between the TEM results and the values of the crystallite size determined by XRD could be related to this fact that the particles size measured through x-ray pattern correlates with size of crystallites and not to the real physical size of particles.

### In-vitro bioactivity studies

Different compositions of commercially available bioactive silicates such as Ceravital, NovaMin and Bioglass (45S5) have been used in clinical applications, successfully. The special surface reactivity causes the formation of an apatite layer on their surface, followed by biological interactions with hard tissue and development of strong bonding with surrounding bone^42^. Surface features of the bioceramics such as roughness, morphology, particle size and porosity, influence their biological behavior^1^. Upon the immersion of the diopside sample in SBF, exchange of Mg^2+^ and Ca^2+^ with hydrogen ions happens. The silica structure collapses due to the loss of ions, which results in the formation of silanol with negative charge on the surface. Subsequently, re-polymerization of silanols forms a silica-rich layer on the diopside surface. This silicon rich layer adsorbs the calcium and phosphate ions and forms a CaO–P_2_O_5_ film. These assembles ions create a calcium phosphate layer on the submerged surface, which then transforms into a calcium deficient carbonate apatite layer, similar to the inorganic part of the natural bone. Consequently, the neighboring tissue can diffuse into this layer and form chemical bonding with the implant surface, providing mechanical support for the tissues^43, 44^.

#### Apatite precipitation on the samples surfaces

Based on our findings represented above, the diopside nano-powders can be simply synthesized via the sol–gel method by using metal salts and metal alkoxide at low temperatures, where no acidic catalysts were used. Confirming the bioactivity of this diopside can open a broad range of applications for it. It has been widely studied that formation of a bone-like apatite layer on the bioactive materials surface is the prerequisite of the binding to living bone in the biological environment^45, 46^. So, the prepared specimens were immersed in SBF for 21 days to study their bioactivity via formation of an apatite layer.

When evaluating bioactivity, it is crucial to take into account variables such as porosity and crystallinity, as higher levels of these factors usually indicate greater bioactivity. As a result, the bioactivity of the diopside powders was studied at a temperature of 1000 °C, which is known to yield highly crystalline powders. Furthermore, prior in vitro studies on the bioactivity of the synthesized β-CaSiO_3_ powders have typically been carried out at temperatures of 1000 °C or above^47, 48^.

**Figure 6:**
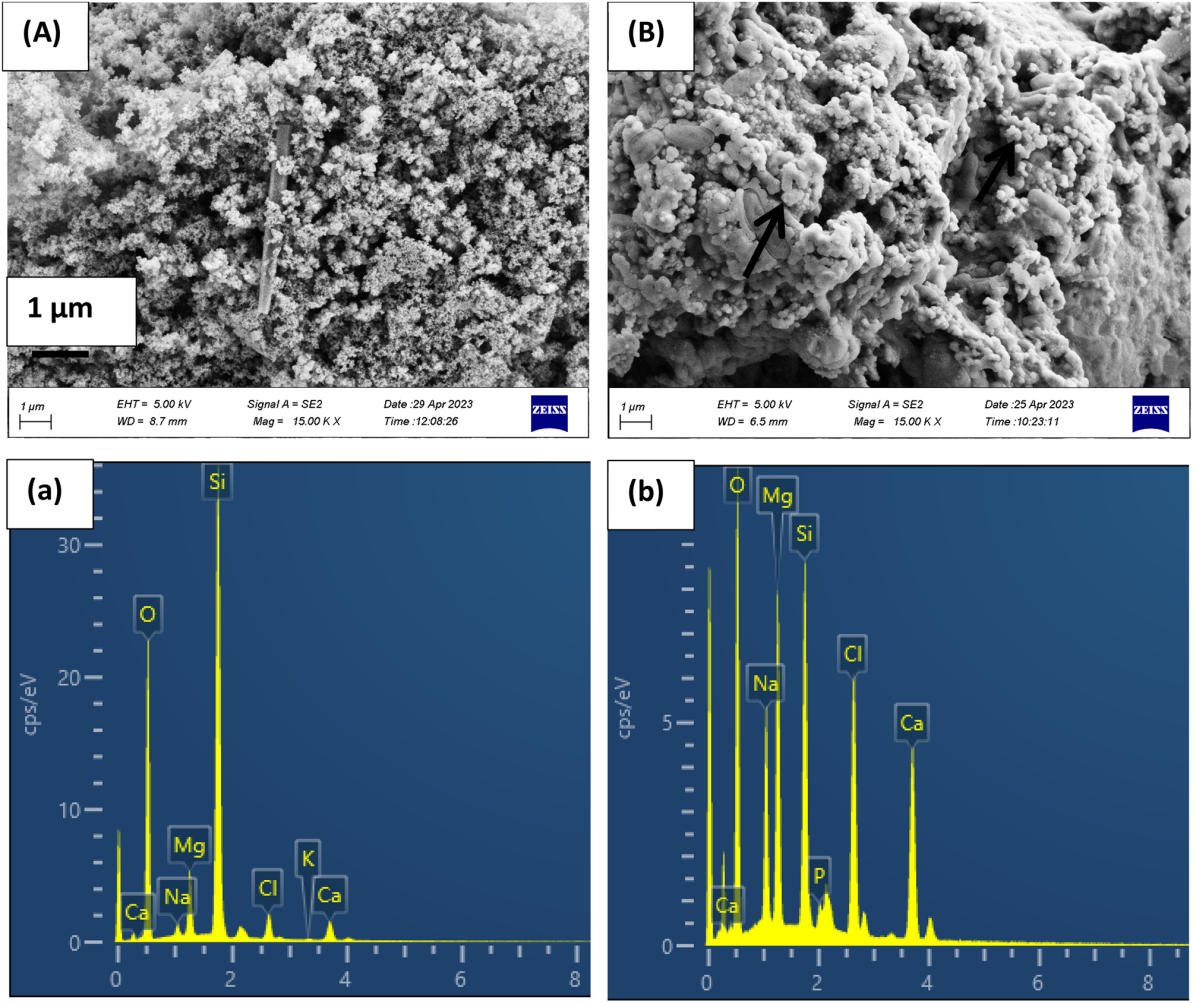
FESEM images and EDX results of the diopside powders after immersion in SBF for 21 days, (**A** and **a**) as-prepared diopside, (**B** and **b**) sintered diopside at 1000 °C.

The surface morphology of the sintered diopside powders was completely changed after the SBF in-vitro test. The nucleated minerals on the surface were clearly observed in white color (Fig. 6B). Based on the EDX examination, the calcium and phosphorus were found in the deposited layer (Fig. 6b). Some bright precipitates could be seen on the surface of the as-prepared diopside (Fig. 6A), but the EDX analysis could not detect the phosphorus on the surface of the non-sintered diopside, as shown in the Table 2. Moreover, the quantity of calcium, phosphorus and other minerals on the surface of the sintered powder was higher than non-sintered one, which reflected the better interactions between sample and SBF medium.

**Table 2:** EDX elemental composition of diopside powder after incubation in SBF solution for 21 days.

Elements	Diopsdie treated at 110 °C after SBF study (Atomic %)	Diopside treated at 1000 °C after SBF study (Atomic %)
O	71.64	64.20
Na	0.87	7.04
Si	20.84	7.25
Ca	1.44	6.17
Cl	1.52	6.37
Mg	3.60	8.43
P	–	0.55

In 1993, Hench showed that phosphate is not an essential part of glass, as phosphorus ions from the electrolyte solution adhere to the glass surface. Phosphate only plays a role in the initial stage of the formation of the calcium phosphate phase^49^. After that, it is released back into the solution due to the degradation of the glass surface. The released calcium and phosphate along with calcium and phosphorus from the SBF combine to create a calcium phosphate-rich layer on the surface of the material, which was resulted in higher concentration of calcium and phosphorus on the surface of the sintered diopside after placing in SBF solution (Fig. 6b) compared to the amorphous one.

**Figure 7:**
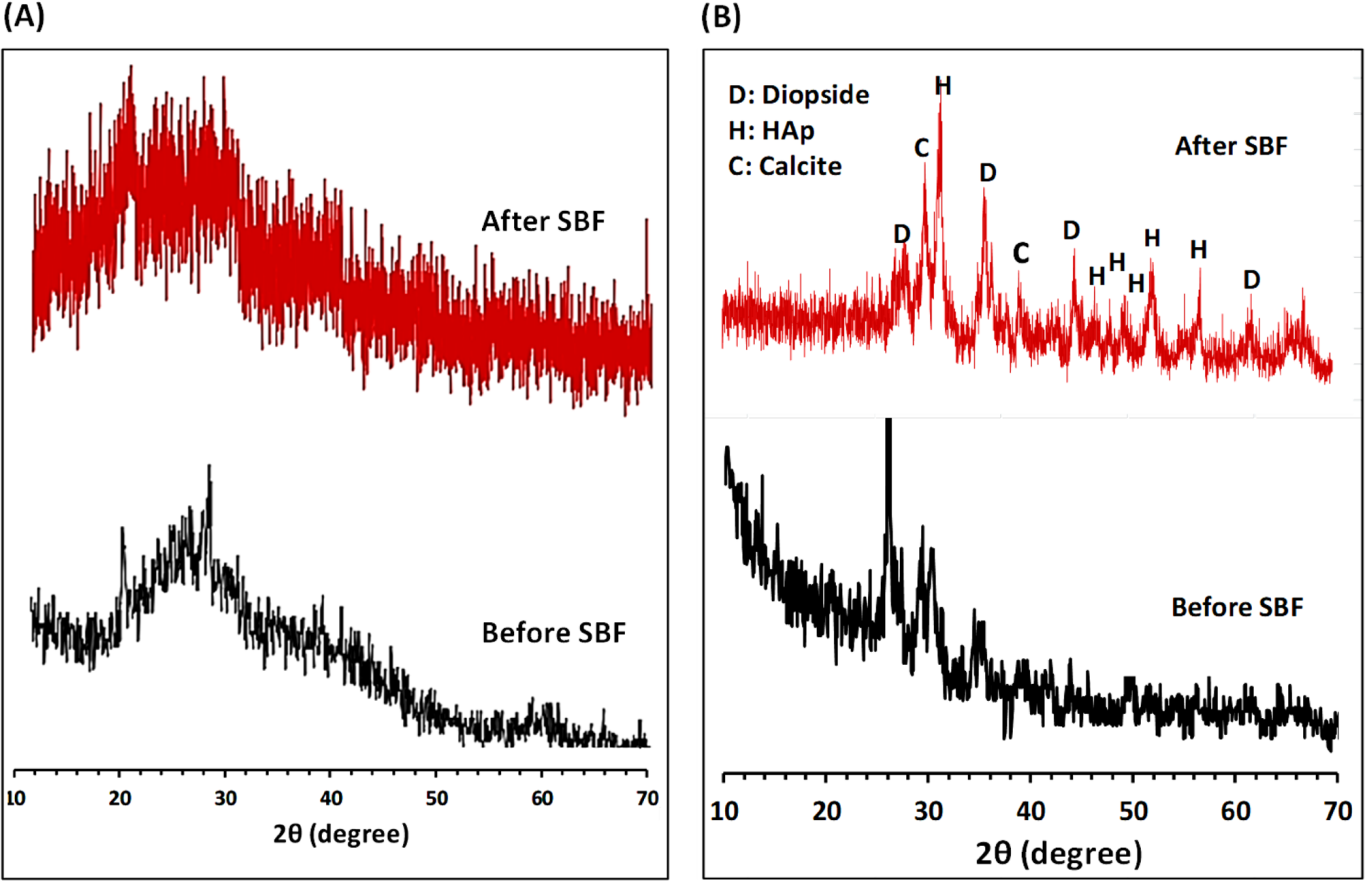
XRD pattern of (**A**) amorphous and (**B**) crystalline diopside, before anf after soaking in SBF solution for 21 days.

In order to scrutinize the precipitated phases, the XRD patterns of the samples were compared before and after placing in SBF, as shown in Fig. 7. Based on the XRD patterns, no apatite formation was observed on the amorphous sample after 21 day of storage in SBF, whereas this layer formed on the crystallized material.

The appeared peaks at 2θ = 31.60, 45.2, 46.6, 48 and 49.43° were found in agreement with ICDD no: 96-900-3550, indicating that the hydroxyapatite layer growth occurred on the sample surface after immersing it in SBF solution. The diffraction intensity of some original diopside peaks decreased and was replaced by hydroxyapatite phase. However, the diopside phase was still detected after 21 days of soaking because of non-homogeneous distribution of hydroxyapatite on the surface of the sample. Trace of calcite (ICDD no: 96-900-1299) was also detected as the minor phase in the X-ray pattern of sample after immersion in SBF, which was in agreement with previous studies^50^. It was demonstrated that the formation of calcium carbonate (commonly reported as calcite crystals) can form on the surface of bioactive glasses as well, instead of or in competition with hydroxyapatite, during in vitro tests. The high release of calcium ions from the glass structure and the presence of hydrogen carbonate ions in the SBF solution allow the precipitation of calcium carbonate^51^.

The microscopic observations (Fig. 6) as well as the XRD results (Fig. 7) confirmed the statement that the crystallization of a bioglass has no inhibiting effect on the formation of an apatite layer. On the contrary, under our test conditions the crystallization had a positive effect on the layer formation.

These observations disclosed the higher bioactivity of the crystalized diopside powders compared to amorphous one. Such observations were also reported by Plewinski et al.^52^ and Leonor et al.^53^. They ascribed these findings to the lower surface energies in crystallized powders that caused the formation of calcium phosphate crystals.

### The drug-carrier analysis

**Figure 8:**
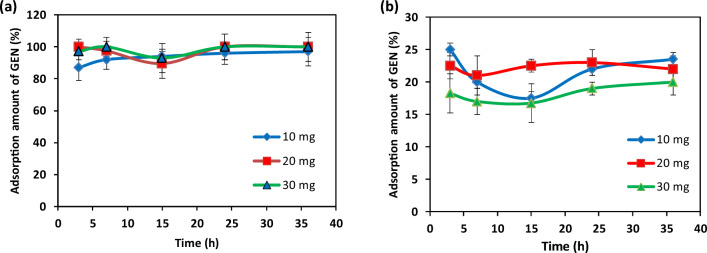
Adsorption profile of GEN on different amount of (**a**) sintered diopside and (**b**) amorphous diopside nanoparticles (n = 3).

The adsorption isotherms of GEN on the surface of the as prepared diopside and also sintered diopside particles at 1000 °C in PBS were shown in Fig. 8. Almost 15–25 wt% of the GEN was adsorbed on the surface of the as-prepared diopside after 3 h of contact, which remain in this range even after 36 h. So, it could be concluded that the drug molecules were attached to the surface of particles rapidly and occupied all free sites of the particles. The observed non-linear pattern of the adsorption process by time (Fig. 8a and b) can be related to desorption of drug molecules during the experiment, which have been attached to the particles again as time passed. In the case of sintered diopside, almost 100% of the drug molecules were adsorbed on the particles surfaces. This high adsorption capacity could be ascribed to the tiny size of the particles that represented high amount of active sites for the attachment of the drug molecules. Higher quantity of particles (30 mg) could also adsorb all drug molecules at the first times of contact. Therefore, it could be concluded that each milligram of the nano powder can adsorb 10 µg of the drug. On the whole, the sintered diopside particles indicated much higher potential for the adsorption of GEN in comparison with the non-sintered diopside. Such observations could be related to the size of the particles, where more agglomerations were formed among the non-sintered diopside particles, which limited the attainable active surface.

Previous reports have observed the interstitial meso-to-micro pores resulting from the escape of water vapors during the transformation of calcium silicate hydrate (C–S–H) into β-CaSiO_3_ at around 800 °C, which formed nodular morphology at a temperature of 1000 °C after complete removal of water vapors^48, 54^. So, in this study, the high capacity of drug adsorption could also be ascribed to the porous structure of the sintered samples, which enable the drug molecules to diffuse inside pores.

Diopside, as an absorbent ceramic, has high capability in attracting the positively charged GEN via the hydroxyl groups. Besides, the adsorption of gentamicin has been reported on the phosphate sites of hydroxyapatite^55^ and on carboxyl groups of collagen^56^.

**Figure 9:**
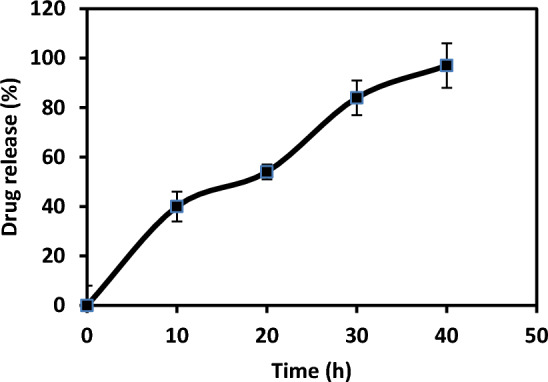
Desorption of GEN from GEN-loaded diopside nanoparticles (n = 3).

The percentage of the desorbed gentamicin sulfate from the drug-loaded diopside powders increased with time, as depicted in Fig. 9. Almost, 79% of the GEN was released within the first day and during the next hours, the entire adsorbed drug was desorbed. However, the burst release of gentamicin from the diopside powders is not suitable for curing diseases in a long period, but in the case of anti-infection property, it can restrict the infection in a short time^55^.

Short and predictable delivery pattern is favorable for medicinal functions of specific drugs such as antibiotics^57^. But strategies to increase the release time up to days or weeks can improve the potential of diopside nanoparticles as a delivery system. A prolonged release may also let better control of the drug release mechanism.

Interestingly, Ahmed El-Fiqi et al.^58^ have reported that around 80% of the loaded ampicillin was released from meso-porous bioglass nanoparticles over 12 h in PBS. It was assumed that ampicillin was sensitive to ion exchange and could not form strong bonds with the bioglass.

## Conclusion

Although numerous studies have reported the preparation of diopside and other bioglasses, but a few number have focused on the sintering behavior, bioactivity and drug carrying potential. Here, a simple and environmentally friendly one-pot sol–gel method was used to successfully synthesize diopside nano-powders. This method offers several benefits, including the use of inexpensive raw materials, mild reaction conditions, a short reaction time, and an easy work-up procedure with no environmental pollution. The crystallization of the dried gel powder and the bioactivity of the sintered body of diopside were studied by immersing it in simulated body fluid (SBF) and examining the effect of thermal treatment.

Based on XRD measurements, the dried gel powder produced using this method was observed to crystallize into a single-phase diopside at 1000 °C, with a higher level of crystallinity than that obtained using the alkoxide method. The heating process generated acidic compounds like HNO_3_ and HCl in the powder, which facilitated the crystallization during thermal treatment. The evaluation of bioactivity demonstrated that a layer rich in crystalline calcium phosphate formed on the surface of crystallized diopside bioglass, indicating that the sintered body of diopside, prepared using the sol–gel process with metal alkoxides and metal salts, without acidic catalysts addition, possess excellent bioactivity, compared with the amorphous powders. This apatite-forming behavior in the SBF suggested that the prepared diopside by using metal salts and metal alkoxide has the potential as a biomedical material. On the other hand, drug loading capacity of the amorphous diopside was compared with the sintered sample for the first time. Sintered diopside nanoparticles could adsorb up to 100% of the gentamicin molecules, as a result of porous structure and high affinity of the powder to the drug. the release study demonstrated that around 80% of the adsorbed GEN was released over the 24 h, resembling a burst release profile.

Despite the disadvantages of the fast release of drug, pharmacologically and economically, burst release may be the ideal strategy of delivery in several occasions. It has been said that a number of drugs require to be administered at varying rates, and for some drugs that employed at the beginning of wound treatment, a primary burst provides instant relief. Recent advances in the potential to target specific cells and organs, permits the location of the delivery to be highly specific, and either burst or prolonged release may be desired at that site^59^.

The morphology of the nanoparticles, initial drug distribution profiles and mechanisms of drug release are all among the aspects which merit further study.

## Data Availability

The data that support the findings of this study are available on request from the corresponding author.
